# Mastering blood gas interpretation: A practical guide for primary care providers

**DOI:** 10.4102/safp.v67i1.6058

**Published:** 2025-04-23

**Authors:** Talat Habib, Arun Nair, Shane Murphy, Hamid Saeed, Nyitiba Ishaya

**Affiliations:** 1 Department of Family Medicine, Robert Mangaliso Sobukwe Hospital, Kimberley, South Africa; 2 Department of Family Medicine, Faculty of Health Sciences, University of the Free State, Bloemfontein, South Africa; 3 Abbey House Medical Centre, Navan, Ireland

**Keywords:** blood gas interpretation, primary care providers, simple acid-base disorders, mixed acid-base disorders, five-step approach, anion gap, delta gap

## Abstract

Accurate arterial blood gas (ABG) interpretation is essential for primary care providers (PCPs), especially in emergency and inpatient settings where timely, informed decisions can significantly impact patient outcomes. This review guides PCPs from basic to advanced interpretation through a systematic five-step approach for ABG analysis, focussing on oxygenation, pH status, and metabolic and respiratory disorders. Emphasising the recognition of complex acid-base disorders that may coexist even when pH appears normal, it incorporates tools such as delta and osmolar gap calculations to address multiple concurrent metabolic disturbances and clarify the interpretation of mixed acid-base conditions. The article also briefly considers the use of arterial and venous blood samples in clinical practice.

## Introduction

Arterial blood gas (ABG) analysis is essential for diagnosing and managing respiratory and metabolic conditions.^1^ Accurate interpretation directly impacts clinical decisions, but can be challenging because of the interplay of various factors.^2^ This article provides step-by-step guidance to enhance primary care providers’ (PCPs) competence in ABG analysis, covering fundamental principles, common pitfalls and strategies for accurate interpretation.

Three primary methods exist for acid-base evaluation: the traditional (Boston), base excess (Copenhagen) and physicochemical (Stewart) methods.^3^ These methods provide a framework for ABG interpretation, guiding the diagnosis and management of acid-base disorders. All three methods generally yield correct clinical interpretation when used properly.^2,3^ Accurate interpretation is crucial to distinguish between simple acid-base disorders – where a primary disturbance and its compensation are present – and mixed disorders, where multiple primary disturbances occur simultaneously.^2,3,4,5^

Proper specimen collection and handling are vital, as errors like non-arterial samples, air bubbles, incorrect anticoagulant levels and delayed analysis can distort results.^2^ When using a liquid heparin syringe, hold it vertically with the needle upward and expel excess heparin and air bubbles.^6^ Air bubbles cause gas exchange with the blood, reducing PaCO_2_ (partial pressure of carbon dioxide) and increasing PaO_2_ (partial pressure of oxygen).^6^ Samples should be placed on ice if immediate analysis is not possible to prevent oxygen metabolism by platelets and leukocytes. Analyse room-temperature samples within 15 min and iced samples within 1 h.^6^ For detailed instructions on safe and accurate arterial blood sampling, refer to guidelines such as that from the World Health Organization (WHO).^7^

Venous blood samples are a practical alternative when arterial access is challenging (Table 1). While less precise for assessing oxygenation and ventilation, venous blood gas (VBG) analysis offers valuable pH and ${\text{HCO}}_{3}^{-}$ (bicarbonate) data and is less invasive, making it useful in conditions like diabetic ketoacidosis or when respiratory involvement is minimal. However, ABG remains essential for accurately evaluating oxygenation and ventilation, particularly in suspected or confirmed respiratory disorders or hypoxaemia.

**TABLE 1:** A comparison of arterial and venous blood gas analyses.

Parameter	ABG analysis	VBG analysis
Purpose	Assess oxygenation status, ventilation, and acid-base balance	Less invasive alternative to ABG; assess acid-base balance and venous CO_2_ levels
Sample site	Radial, brachial or femoral artery	Peripheral vein in the arm
Key parameters (Normal values)	pH: 7.35–7.45 (H^+^ 35–45 nmol/L)	pH: 7.32–7.43 (H^+^ 37–48 nmol/L)
	PaO_2_: 80–100 mmHg (10.6–13.3 kPa)	PvO_2_: 25–40 mmHg (3.3–5.3 kPa)
	PaCO_2_: 35–45 mmHg (4.7–6.0 kPa)	PvCO_2_: 41–50 mmHg (5.5–6.7 kPa)
	${\text{HCO}}_{3}^{-}$: 22–26 mmol/L	${\text{HCO}}_{3}^{-}$: 23–27 mmol/L
	BE: -2 to +2 mmol/L	BE: Similar to arterial values
Limitations	Invasive and painful procedure. Risk of complications such as bleeding, haematoma and arterial injury	Less accurate for assessing oxygenation status, may not detect hypoxaemia. Differences in pH, PvO_2_ and PvCO_2_ require careful interpretation

ABG, arterial blood gas; VBG, venous blood gas; pH, potential of hydrogen; PaO_2_, partial pressure of oxygen in arterial blood; PaCO_2_, partial pressure of carbon dioxide in arterial blood; HCO_3_, bicarbonate; PvO_2_, partial pressure of oxygen in venous blood; PvCO_2_, partial pressure of carbon dioxide in venous blood; BE, base excess.

## Starting the interpretation

To interpret an ABG report accurately, begin by verifying the internal consistency of the ABG values and obtaining relevant clinical information before applying the five-step approach.

### Verify the consistency of the arterial blood gas report

Checking the internal consistency of ABG reports is essential to ensure that the measured parameters align with known physiological relationships.^8^ This practice helps identify discrepancies that may arise from preanalytical and analytical errors. Internal consistency differs from calibration: calibration ensures that the machine provides accurate measurements, while internal consistency confirms that the reported values are logical and cohesive based on physiological norms. Common issues affecting internal consistency include improper sample collection, delays in processing and contamination. The *Henderson-Hasselbalch equation* verifies ABG consistency by relating pH, ${\text{HCO}}_{3}^{-}$ and PaCO_2_^9^:


$$\text{pH}=6.1+\log\;({\text{HCO}}_{3}^{-}/0.03\times {\text{PaCO}}_{2})$$ [Eqn 1]

*6.1* is the acid dissociation constant (pKₐ) for carbonic acid at body temperature, and *0.03* is the solubility coefficient of CO_2_ in the blood (mmol/L per mmHg).

The equation requires a calculator with a logarithm function, available on most mobile devices. See Box 1 for a practical example. Alternatively, embedding the equation in a spreadsheet allows for automatic calculation of pH upon entering the values of ${\text{HCO}}_{3}^{-}$ and PaCO_2_. If the calculated pH differs from the reported pH by more than ± 0.05, suspect an error in one or more parameters and repeat the test, as such discrepancies are common.^9^

BOX 1 Clinical case study – Application of the five-step approach to arterial blood gas interpretation.

**Clinical scenario:**

A 55-year-old man is brought to the emergency department with altered mental status, nausea, and vomiting. He has a history of alcoholism and was found near empty antifreeze (ethylene glycol) containers.

**Vital signs:**

Pulse: 110 bpm, BP: 90/60 mmHg, RR: 28 breaths/min, SpO₂: 94% on room air

**Exam:**

Lethargic, Kussmaul respirations, dehydrated

**Laboratory results:**

-ABG: pH: 7.12, PaCO₂: 22 mmHg, PaO₂: 85 mmHg, ${\text{HCO}}_{3}^{-}$: 7 mmol/L
- Electrolytes: Na⁺: 140 mmol/L, Cl⁻: 100 mmol/L, Serum Osmolality: 360 mOsm/kg, Serum Albumin: 40 g/L

**Starting the interpretation**

*Verify ABG report’s internal consistency*

- Method 1: Henderson-Hasselbalch Equation: pH = 6.1 + log (${\text{HCO}}_{3}^{-}$ / 0.03 × PaCO₂) = 6.1 + log (7/0.03 × 22) = 6.1 + log (7/0.66) = 7.125
- Calculated pH matches reported pH; ABG values are consistent.

*Obtain clinical information*

- History suggests ethylene glycol ingestion.
- Symptoms indicate metabolic acidosis.

**Five-step approach**

**Step 1:** Oxygenation status

- PaO₂: 85 mmHg on room air (within normal range)
- Expected PaO₂: 21% × 5 ≈ 100 mmHg.
- Age adjusted PaO₂ = 100 mmHg – (0.3 × Age in years) = 83.5 mmHg
- Interpretation: No significant hypoxaemia.

**Step 2:** pH status

- pH 7.12: Indicates acidaemia.

**Step 3:** Determine primary disorder

- Low ${\text{HCO}}_{3}^{-}$ (7 mmol/L): Primary metabolic acidosis.
- Low PaCO₂ (22 mmHg): Respiratory compensation.
- Calculate Expected PaCO₂ (Winter’s Formula): Expected PaCO₂ = (1.5 × ${\text{HCO}}_{3}^{-}$) + 8 ± 2 = (1.5 × 7) + 8 ± 2 = 18.5 ± 2 mmHg
- Expected range: 16.5–20.5 mmHg

Measured PaCO₂ (22 mmHg) is slightly above expected range; suggests inadequate respiratory compensation or concurrent respiratory acidosis.

**Step 4:** Calculate Anion Gap (AG)

- AG: (140 – [100 + 7]) = 33 mmol/L

Elevated AG indicates high anion gap metabolic acidosis (HAGMA).

- Calculate Osmolar Gap (OG):

Calculated Osmolality = (2 × 140) + 6 + 5 = 291 mOsm/kg

OG = measured serum osmolality – calculate serum osmolality = 360 – 291 = 69 mOsm/kg

Elevated OG suggests ethylene glycol poisoning.

**Step 5:** Assess for additional disorders (Δ Gap)

- ΔAG = 33 – 12 = 21 mmol/L
- Δ${\text{HCO}}_{3}^{-}$ = 24 – 7 = 17 mmol/L
- Δ Gap = ΔAG – Δ${\text{HCO}}_{3}^{-}$ = 21 – 17 = +4 mmol/L

Delta gap is within normal range (−6 to +6); no additional acid-base disorder is revealed.

**Conclusion**

- Primary disorder: High anion gap metabolic acidosis because of ethylene glycol poisoning.
- Compensation: Inadequate respir atory compensation; possible concurrent respiratory acidosis.

ABG, arterial blood gas; pH, potential of hydrogen; HCO_3_, bicarbonate.

### Obtain clinical information

Gather focussed clinical history and examination findings to contextualise ABG results.^2,9^ Determining whether an ABG abnormality is acute or chronic informs the urgency and type of treatment. For example, chronic respiratory acidosis may not require immediate intervention compared to acute cases. Serial blood gas measurements can monitor the patient’s response to treatment and disease progression.

### A five-step approach to ABG interpretation: The CLEAR path

**FIGURE 1:**
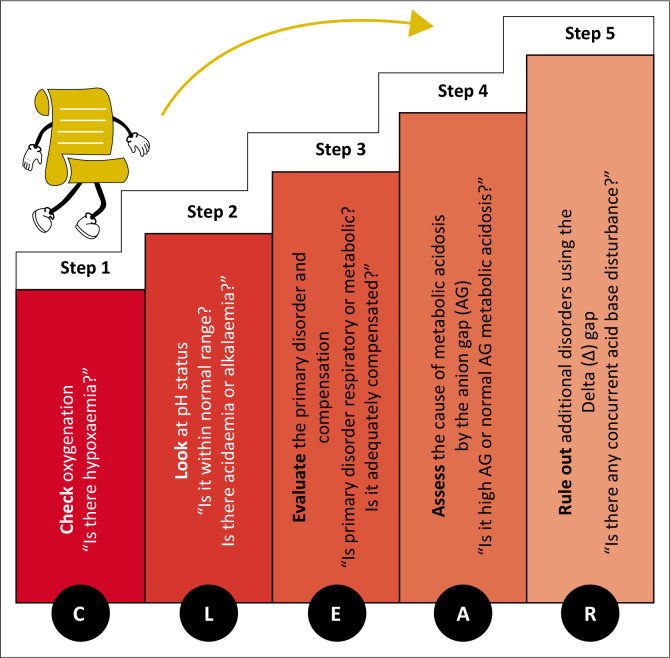
The CLEAR path.

**FIGURE 2:**
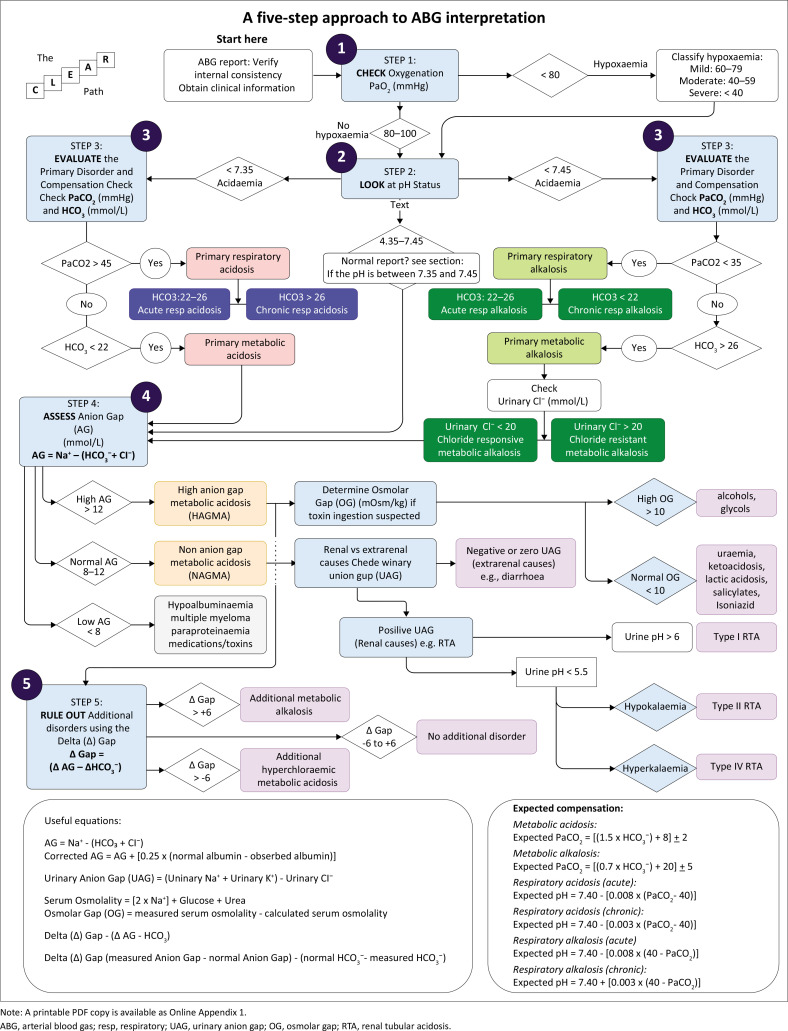
A five-step approach to arterial blood gas interpretation.

The ‘CLEAR’ mnemonic provides a structured five-step framework for ABG interpretation (Figure 1) with a flow diagram (Figure 2) offering a visual summary.

#### Step 1: *Check* oxygenation

- Check partial pressure of oxygen (PaO_2_)

*Normal PaO*_*2*_ *(80 mmHg – 100 mmHg):* Proceed to step 2.

*Decreased PaO*_*2*_ *(< 80 mmHg):* Suggests hypoxaemia. It should be interpreted in the context of fraction of inspired oxygen (FiO_2_). Estimate the expected PaO_2_ by multiplying FiO_2_ by 5, and use this to classify hypoxaemia as mild, moderate or severe:^2^

- Calculate expected PaO_2_:

$$\text{Expected }{\text{PaO}}_{2}={\text{FiO}}_{2}\%\times 5$$ [Eqn 2]

Age adjusted PaO_2_ on room air = 100 mmHg – (0.3 × age in years).

- Classify hypoxaemia:
  *PaO*_*2*_ *60 mmHg – 79 mmHg:* Mild hypoxaemia
  *PaO*_*2*_ *40 mmHg – 59 mmHg:* Moderate hypoxaemia
  *PaO*_*2*_ *< 40 mmHg:* Severe hypoxaemia

#### Step 2: *Look* at pH status

- If the pH is between 7.35 and 7.45

Check if other parameters are within normal range (Table 1). A normal pH, PaCO_2_ and ${\text{HCO}}_{3}^{-}$ does not exclude acid-base disorders.^10^ If anion gap (AG) is increased, a patient can still have metabolic acidosis and alkalosis. When all the parameters, including AG, are within normal limits, the patient has a normal acid-base balance.

A normal pH with abnormal PaCO_2_ and ${\text{HCO}}_{3}^{-}$ indicates a mixed acid-base disorder, with acidosis and alkalosis balancing each other.^5^ Calculating the AG is essential in ABG interpretation to uncover hidden acid-base disorders.^11^

- Acidaemia or alkalaemia?
  ■ pH < 7.35: Acidaemia, suggesting either respiratory acidosis, metabolic acidosis or a combination. Proceed to Step - For acidaemia (pH < 7.35).
  ■ pH > 7.45: Alkalaemia, suggesting either respiratory alkalosis, metabolic alkalosis or a combination. Proceed to Step - For alkalaemia (pH > 7.45).

#### Step 3: *Evaluate* the Primary Disorder and Compensation

- For acidaemia (pH < 7.35):

Check PaCO_2_ – *Elevated PaCO*_*2*_ *(> 45 mmHg):* Suggests *Primary respiratory acidosis.* For example, if an ABG report shows a pH of 7.30, PaCO2 of 50 mmHg, and ${\text{HCO}}_{3}^{-}$ of 24 mmol/L, this indicates acidaemia. The elevated PaCO2 suggests that the primary cause is respiratory acidosis:

- *Assess ${\text{HCO}}_{3}^{-}$ levels*:
  ■ Normal ${\text{HCO}}_{3}^{-}$ (22 mmol/L – 26 mmol/L): Likely *acute respiratory acidosis* (no time for renal compensation).
  ■ Elevated ${\text{HCO}}_{3}^{-}$ (> 26 mmol/L): Indicates *chronic respiratory acidosis* (renal compensation has occurred).
- *Calculate expected renal compensation*:
  ■ Refer to Table 2 for expected compensation values and determine if there is an additional metabolic disorder.

**TABLE 2:** Primary acid-base disorders and expected compensation.

Primary acid-base disorders	pH	${\text{HCO}}_{3}^{-}$	PaCO_2_	Expected compensation
**Metabolic**				
Acidosis	↓	↓		Expected PaCO_2_ = [(1.5 × ${\text{HCO}}_{3}^{-}$) + 8] ± 2 Less precise: Expected PaCO_2_ = last two digits of pH If PaCO_2_ > expected: Concomitant respiratory acidosis If PaCO_2_ < expected: Concomitant respiratory alkalosis
Alkalosis	↑	↑		Expected PaCO_2_ = [(0.7 × ${\text{HCO}}_{3}^{-}$) + 20] ± 5 If PaCO_2_ > expected: Concomitant respiratory acidosis If PaCO_2_ < expected: Concomitant respiratory alkalosis
**Respiratory**				
Acidosis				
Acute^†^	↓		↑	Expected pH = 7.40 – [0.008 × (PaCO_2_ – 40)] ${\text{HCO}}_{3}^{-}$ will increase by 1 mmol/L for each 10 mmHg rise in PaCO_2_ above 40 mmHg
Chronic^‡^	↓		↑	Expected pH = 7.40 – [0.003 × (PaCO_2_ – 40)] ${\text{HCO}}_{3}^{-}$ will increase by 4 mmol/L for each 10 mmHg rise in PaCO_2_ above 40 mmHg
Alkalosis				
Acute^†^	↑		↓	Expected pH = 7.40 + [0.008 × (40 – PaCO_2_)] ${\text{HCO}}_{3}^{-}$ will decrease by 2 mmol/L for each 10 mmHg decrease in PaCO_2_ below 40 mmHg
Chronic^‡^	↑		↓	Expected pH = 7.40 + [0.003 × (40 – PaCO_2_)] ${\text{HCO}}_{3}^{-}$ will decrease by 5 mmol/L for each 10 mmHg decrease in PaCO_2_ below 40 mmHg

pH, potential of hydrogen; PaCO_2_, partial pressure of carbon dioxide in arterial blood; ${\text{HCO}}_{3}^{-}$, serum bicarbonate concentration; mmol/L, millimoles per litre.

↑, increased; ↓, decreased.

† Acute < 3–5 days;

‡ , Chronic > 3–5 days.

Check HCO_3_ – *Decreased ${\text{HCO}}_{3}^{-}$ (< 22 mmol/L):* Suggests *Primary metabolic acidosis:*

- *Calculate expected respiratory compensation*:
  ■ Expected PaCO_2_ = [(1.5 × ${\text{HCO}}_{3}^{-}$) + 8] ± 2 (Table 2). For example, if measured ${\text{HCO}}_{3}^{-}$ is 12 mmol/L, then expected PaCO_2_ will be [(1.5 × 12) + 8] ± 2 = 26 ± 2 mmHg. If the patient’s actual PaCO_2_ is within 24 mmHg – 28 mmHg, compensation is appropriate. Values outside this range suggest a mixed disorder.
- *Proceed to Step 4*.
- For alkalaemia (pH > 7.45):

Check PaCO_2_ – *Decreased PaCO*_*2*_ *(< 35 mmHg):* Suggests *Primary respiratory alkalosis*:

- *Assess ${\text{HCO}}_{3}^{-}$ levels*:
  ■ Normal ${\text{HCO}}_{3}^{-}$ (22 mmol/L – 26 mmol/L): Likely *acute respiratory alkalosis.*
  ■ Decreased ${\text{HCO}}_{3}^{-}$ (< 22 mmol/L): indicates *chronic respiratory alkalosis* (renal compensation).
- *Calculate expected renal compensation*:
  ■ Refer to Table 2 for expected compensation values and determine if there is an additional metabolic disorder.

Check *HCO*_*3*_ *– Elevated ${\text{HCO}}_{3}^{-}$ (> 26 mmol/L):* Suggests *Primary metabolic alkalosis:*

- *Calculate expected respiratory compensation*:
  ■ Expected PaCO_2_ = [(0.7 × ${\text{HCO}}_{3}^{-}$) + 20] ± 5 (Table 2). For example, if measured ${\text{HCO}}_{3}^{-}$ is 12 mmol/L, then expected PaCO_2_ will be [(0.7 × 12) + 20] ± 5 = 28.4 ± 5 mmHg. If the patient’s actual PaCO_2_ is within 23.4 mmHG – 33.4 mmHg, compensation is appropriate. Values outside this range suggest a mixed disorder.
- *Classify metabolic alkalosis* – *Metabolic alkalosis is divided into two groups based on urinary chloride:*
  ■ *Chloride responsive*: (extravascular volume depletion because of vomiting, chronic diarrhoea, diuretic, post-hypercapnic).
    Urinary chloride < 20 mmol/L. These patients respond to administered sodium chloride (NaCl) infusion.
  ■ *Chloride resistant*: Urinary chloride > 20 mmol/L; typically seen in euvolemic or fluid overload states. These patients do not respond to NaCl infusion and need potassium chloride (KCl) to correct hypokalaemia. The condition often results from excessive mineralocorticoids, causing sodium retention and potassium excretion.

#### Step 4: *Assess* the cause of metabolic acidosis by the anion gap (AG)

- Calculate the anion gap.

*AG* = *Na*^+^ – (${\text{HCO}}_{3}^{-}$ + *Cl*^−^)

Normal AG range: 8 mmol/L – 12 mmol/L

- Adjust for Hypoalbuminaemia:

To correct the AG for hypoalbuminaemia, add 0.25 mmol/L to the AG for each 1 g/L drop in albumin below 40 g/L.^10^

*Corrected AG = AG + 0.25 (normal albumin – observed albumin)*

- Interpret the anion gap.

High anion gap (> 12 mmol/L) This indicates *High Anion Gap Metabolic Acidosis* (HAGMA):

- AG is increased because of retention of unmeasured anion from the titrated strong acid. Bicarbonate is reduced through buffering of added strong acid.
- If AG is ≥ 20 mmol/L, then a *metabolic acidosis* is present *regardless* of the pH or serum ${\text{HCO}}_{3}^{-}$ values.
- Determine *Osmolar Gap* (*OG*), if toxin ingestion is suspected. Osmolar Gap helps identify the presence of unmeasured osmotically active substances, for example alcohols, glycols, in the blood.
*Osmolar Gap = measured serum osmolality – calculate serum osmolality*
  *Serum Osmolality* = 2 × Na + Glucose + Urea
  *High OG* (> 10 mOsm/kg): methanol, ethylene glycol
  *Normal OG* (< 10 mOsm/kg): Uraemia, ketoacidosis, lactic acidosis, salicylates, isoniazid
- Proceed to Step 5.

Normal anion gap (8 mmol/L – 12 mmol/L) This suggests *Non-Anion Gap Metabolic Acidosis* (NAGMA) (Hyperchloremic metabolic acidosis):

- Mainly because of losses of bicarbonate (commonly from gastrointestinal tract), and/or increased chloride ingestion, and/or infusion of substances that release hydrochloric acid (such as NaCl).
- No anion gap is present because of the absence of unmeasured anion from titrated acid and secondary chloride retention with ${\text{HCO}}_{3}^{-}$ loss. Kidneys fail to reabsorb or regenerate ${\text{HCO}}_{3}^{-}$.
- Differentiate renal from extrarenal causes in NAGMA –The urinary anion gap is used to differentiate renal from extrarenal causes.
   *Urinary anion gap* (UAG) = (Urinary Na^+^ + Urinary K^+^) – Urinary Cl^–^
   *A negative or zero UAG* indicates an extrarenal cause, such as diarrhoea.
   *A positive UAG* suggests a renal cause, such as renal tubular acidosis (RTA).
  ■ A *positive UAG* with urine *pH of > 6* is suggestive of *Type I RTA*
  ■ A *positive UAG* with urine pH of < 5.5 with hypokalaemia is suggestive of *Type II RTA*; and with *hyperkalaemia* indicates *Type IV RTA*.

Low AG (< 8 mmol/L)^12^:

- Check for hypoalbuminaemia.
- Assess for multiple myeloma and paraproteinaemia: Consider serum protein electrophoresis.
- Evaluate electrolytes: Look for hypercalcaemia or hypermagnesemia.
- Review medications and toxins: Consider lithium toxicity; exposure to bromide or iodide.

#### Step 5: *Rule out* Additional Disorders using the Delta (Δ) Gap

- The Δ Gap is a calculation used to uncover additional acid-base disorders in the context of HAGMA.^11,13^ It compares the change (Δ) in the AG to the change (Δ) in ${\text{HCO}}_{3}^{-}$ levels.
   Δ *Gap* = (Δ *AG* – Δ *${\text{HCO}}_{3}^{-}$*)
   Δ *Gap* = (Measured Anion Gap – Normal Anion Gap) − (Normal ${\text{HCO}}_{3}^{-}$ − Measured ${\text{HCO}}_{3}^{-}$)
   Δ *Gap* = (AG-12) – (24-${\text{HCO}}_{3}^{-}$)
   For every 1 mmol/L rise in AG, ${\text{HCO}}_{3}^{-}$ should drop by 1 mmol/L in a simple acid-base disorder.
   Δ Gap: –6 to +6 (normal) No additional disorder.
   Δ Gap > +6 suggests an additional metabolic alkalosis, since the rise in AG is more than the fall in ${\text{HCO}}_{3}^{-}$.
   Δ Gap < –6 suggests an additional hyperchloremic metabolic acidosis, because the rise in AG is less than the fall in ${\text{HCO}}_{3}^{-}$.
- Up to three disorders can coexist, and Δ Gap calculations can help identify them. While two metabolic abnormalities can coexist, only one respiratory disorder can occur at a time, as a patient cannot simultaneously have both hypoventilation and hyperventilation.^14^

See Box 1 for a practical example of this five-step approach in practice.

## Recommendations

- Obtain ABG and/or VBG measurements when clinically indicated, interpret them confidently and adhere to a structured, stepwise approach.
- The printable PDF flowchart (supplementary file) with this article provides a quick-reference tool in clinical settings.
- Use apps and online calculators (e.g. MDCalc) for quick ABG analysis. Investigate emerging AI-driven tools while validating their outputs against clinical judgement.
- Reflect on challenging cases to identify learning opportunities and share with colleagues to promote collective learning.

## Conclusion

Blood gas interpretation is crucial for PCPs, enabling timely, impactful decisions. Mastering ABG fundamentals and using a systematic approach help clinicians confidently manage acid-base disorders. Practical knowledge and awareness of common pitfalls enhance diagnostic skills, empowering PCPs to deliver high-quality care. Ongoing learning, collaboration and reflective practice keep PCPs at the forefront of evolving healthcare.

## References

[1] Dzierba AL, Abraham P. A practical approach to understanding acid–base abnormalities in critical illness. J Pharm Pract. 2011;24(1):17–26. 10.1177/0897190010388153 21507871

[2] Sood P, Paul G, Puri S. Interpretation of arterial blood gas. Indian J Crit Care Med. 2010;14(2):57. 10.4103/0972-5229.68215 20859488 PMC2936733

[3] Emmett M. Stewart’s textbook of acid–base. Kidney Int. 2009;75(12):1247–1248. 10.1038/ki.2009.148

[4] Sirker AA, Rhodes A, Grounds RM, Bennett ED. Acid-base physiology: The ‘traditional’ and the ‘modern’ approaches. Anaesthesia. 2002;57(4):348–356. 10.1046/j.0003-2409.2001.02447.x 11939993

[5] Mane A. Mixed acid-base disorders. In: Mane A, editor. Arterial blood gas interpretation in clinical practice. Cham: Springer International Publishing, 2021; p. 97–114.

[6] Worcestershire Acute Hospitals NHS Trust. Arterial blood gas sampling for trained professionals [homepage on the Internet]. Worcestershire Acute Hospitals NHS Trust; 2023 [cited 2025 Jan 19]. Available from: https://apps.worcsacute.nhs.uk/KeyDocumentPortal/Home/DownloadFile/3780

[7] World Health Organization. WHO guidelines on drawing blood: Best practices in phlebotomy. Geneva: World Health Organization; 2010. 23741774

[8] Kaufman DA, Society AT. Interpretation of arterial blood gases (ABGs). New York: American Thoracic Society Section of Pulmonary & Critical Care Medicine; 2016.

[9] Rodríguez-Villar S, Do Vale BM, Fletcher HM. The arterial blood gas algorithm: Proposal of a systematic approach to analysis of acid-base disorders. Rev Esp Anestesiol Reanim. 2020;67(1):20–34. 10.1016/j.redar.2019.04.001 31826801

[10] Barletta JF, Muir J, Brown J, Dzierba A. A systematic approach to understanding acid-base disorders in the critically ill. Ann Pharmacother. 2023;58(1):65–75. 10.1177/10600280231165787 37125739

[11] Mavrothalassitis O, Thind BS, Agrawal A. Four acid-base disturbances in a critically-ill patient undergoing emergent abdominal surgery. Case Rep Crit Care. 2022;2022(1):1285598. 10.1155/2022/1285598 35836728 PMC9273465

[12] Kraut JA, Madias NE. Serum anion gap: Its uses and limitations in clinical medicine. Clin J Am Soc Nephrol. 2007;2(1):162–174. 10.2215/CJN.03020906 17699401

[13] Wrenn K. The delta (Δ) gap: An approach to mixed acid-base disorders. Ann Emerg Med. 1990;19(11):1310–1313. 10.1016/S0196-0644(05)82292-9 2240729

[14] Hasan A. pH. In: Hasan A, editor. Handbook of blood gas/acid-base interpretation. London: Springer, 2013; p. 143–163.

